# Comprehensive SPME-GC-MS Analysis of VOC Profiles Obtained Following High-Temperature Heating of Pork Back Fat with Varying Boar Taint Intensities

**DOI:** 10.3390/foods10061311

**Published:** 2021-06-07

**Authors:** Clément Burgeon, Alice Markey, Marc Debliquy, Driss Lahem, Justine Rodriguez, Ahmadou Ly, Marie-Laure Fauconnier

**Affiliations:** 1 Laboratory of Chemistry of Natural Molecules, Gembloux Agro-Bio Tech, Université de Liège, Passage des Déportés 2, 5030 Gembloux, Belgium; Alice.Markey@student.uliege.be (A.M.); marie-laure.fauconnier@uliege.be (M.-L.F.); 2 Service de Science des Matériaux, Faculté Polytechnique, Université de Mons, Rue de l’Epargne 56, 7000 Mons, Belgium; marc.debliquy@umons.ac.be (M.D.); Justine.RODRIGUEZ@umons.ac.be (J.R.); 3 Materia Nova ASBL, Materials R&D Centre, Parc Initialis, Avenue Nicolas Copernic 3, 7000 Mons, Belgium; driss.lahem@materianova.be (D.L.); ahmadou.ly@materianova.be (A.L.)

**Keywords:** back fat, boar taint, entire male pig, GC-MS, lipid oxidation, meat quality, pork meat, SPME, VOC

## Abstract

Boar taint detection is a major concern for the pork industry. Currently, this taint is mainly detected through a sensory evaluation. However, little is known about the entire volatile organic compounds (VOCs) profile perceived by the assessor. Additionally, many research groups are working on the development of new rapid and reliable detection methods, which include the VOCs sensor-based methods. The latter are susceptible to sensor poisoning by interfering molecules produced during high-temperature heating of fat. Analyzing the VOC profiles obtained by solid phase microextraction gas chromatography–mass spectrometry (SPME-GC-MS) after incubation at 150 and 180 °C helps in the comprehension of the environment in which boar taint is perceived. Many similarities were observed between these temperatures; both profiles were rich in carboxylic acids and aldehydes. Through a principal component analysis (PCA) and analyses of variance (ANOVAs), differences were highlighted. Aldehydes such as (*E*,*E*)-nona-2,4-dienal exhibited higher concentrations at 150 °C, while heating at 180 °C resulted in significantly higher concentrations in fatty acids, several amide derivatives, and squalene. These differences stress the need for standardized parameters for sensory evaluation. Lastly, skatole and androstenone, the main compounds involved in boar taint, were perceived in the headspace at these temperatures but remained low (below 1 ppm). Higher temperature should be investigated to increase headspace concentrations provided that rigorous analyses of total VOC profiles are performed.

## 1. Introduction

Nowadays, a top priority for the pork industry is being able to correctly discriminate tainted from untainted boar carcasses. In fact, boar taint is a strong and unpleasant smell found in the meat of some uncastrated male pigs. This smell appears upon cooking of boar tainted meat and is due to the release of a complex mixture of molecules. The major molecules responsible for this smell are the steroid androstenone (5-α-androst-16-en-3-one) and the tryptophan metabolite skatole (3-methylindole), which are well-known for their urine and fecal smell, respectively [1,2].

To prevent the development of such molecules, surgical castration without pain relief has often been used worldwide given that it is a fast, cheap, and handy castration technique for farmers. However, this practice has been criticized for the pain and stress that it inflicts to piglets. Hence, alternatives to surgical castration have been suggested and are now being promoted [3]. Out of all, two castration techniques appear more realistic: immunocastration (i.e., testicular functions are deactivated through the neutralization of the hypothalamic–pituitary–gonadal axis hormones [4]) and rearing of entire males. Whether it is to discriminate tainted uncastrated male pigs or simply to ensure that immunocastration has functioned correctly, the detection of tainted carcasses is an essential step in the slaughtering process.

Currently, many research studies are taking place to develop new detection methods that are ideally low cost (less than 1.30 euro/analysis), fast (less than 10 s/analysis), 100% specific and sensitive (no false negatives and no false positives), and automated [5]. These criteria are essential for methods to be used on the slaughter line.

Several detection principles have been investigated throughout the years [6]. Mass spectrometry-based methods have recently been examined and have shown interesting results. Rapid evaporative ionization mass spectrometry (REIMS) provided highly accurate classification of tainted and untainted samples at a fast speed and has shown its potential to be used for online applications given its hand-held sampling tool and estimated low cost [7]. Laser diode thermal desorption–tandem mass spectrometry (LDTD-MS/MS) has also been thoroughly investigated [8,9,10]. This method achieved good validation criteria, fast analysis (once sample preparation has been performed, analysis in itself takes less than 10 s/sample), and is currently being tested in a Danish slaughterhouse [11]. However, both methods would require substantial investment (expensive instruments and need for skilled staff), which could lead to reluctance in their application.

Other methods recently tested and presenting lower investment cost are devices based on Raman spectroscopy [12,13] and a new specific sensor system based on screen-printed carbon electrodes [14,15]. Additionally, these techniques are easy to use given the hand-held measuring tool. However, both still need further validation given high prediction errors for Raman spectroscopy and the absence of real slaughterhouse testing with the sensor system.

The rapid detection of boar taint through volatile organic compounds (VOCs) detection has also been widely studied. Some researchers have tried using gas chromatography mass spectrometry (GC-MS) for this purpose [16,17]. However, the high initial investment and long (i.e., minimum 3.5 min [16]) analysis time remained two main drawbacks of GC-MS methods.

Boar taint detection through the use of e-noses has been extensively studied some years ago [18,19,20,21,22]. An e-nose is composed of an array of sensors for which a response is induced when gases, and in this case VOCs, are perceived at their surface. In a recent review by Burgeon et al. (2021) [6], the great potential of new sensor material for skatole and androstenone has been discussed, and this review concluded that sensor-based methods might be a solution for the rapid slaughterhouse detection of boar taint provided that it is able to detect low headspace concentrations of skatole and androstenone in a VOCs-rich environment. This working environment is due to the extraction conditions used to volatilize skatole and androstenone.

In fact, skatole and androstenone are lipophilic molecules with low vapor pressure (7.3 × 10^−4^ kPa and 1.3 × 10^−6^ kPa at 25 °C, respectively); hence, fat must be heated at high temperatures to allow the volatilization of these molecules. This heating leads to the release of a variety of molecules. Most of these molecules are products of lipids degradation (oxidation of fatty acids starting at 70 °C [23]). Lipids can oxidize in three main ways: autoxidation, enzymatic-catalyzed oxidation, and photo-oxidation. However, the most probable oxidation mechanism during fat heating remains autoxidation where the unsaturated fatty acids react with oxygen, which is activated by temperature in this case, to produce free radicals. These free radicals are unstable and therefore decompose to form various molecules, including acids, alcohols, esters, ketones hydrocarbons, and aldehydes. The latter are present in significant quantities in products that underwent oxidation processes [24].

Such a VOCs-rich environment can quickly lead to sensor poisoning, i.e., binding of VOCs to the sensor’s surface, and in turn lead to temporal sensor drift. Such drift is defined as the gradual deviation of the sensor’s response when exposed to the same molecule in the same environment [25]. Understanding the VOCs environment in which the volatilization of skatole and androstenone takes place is primordial, as this could help in creating new drift-reduction solutions, which are physical solutions (such as filters) aiming to reduce interfering VOCs present in the headspace but also creating more robust drift correction models taking such environments into account.

Until now, none of the above-mentioned methods have stood out compared to the others, and that is why current slaughterhouse boar taint detection is still performed either through a colorimetric method [26] or mainly by sensory evaluation [27].

Hence, the objective of this research was to examine elevated temperature VOC profiles to facilitate new sensor development, gain the understanding of VOCs perceived during boar taint sensory evaluations, and lastly help in understanding which VOCs perceived by the consumers during the cooking of pork meat are lipid-derived. The 150 and 180 °C temperatures were used in the current study, as they are frequently encountered for sensory evaluation in the frame of boar taint detection [28,29,30,31,32] and appear in the range of temperatures used for cooking by consumers [33].

Rius et al. (2005) [34] have already analyzed VOCs produced when heating fat at a temperature of 120 °C. However, only back fat with low concentrations in skatole and androstenone was analyzed, and comparisons of heating temperatures were not performed.

To the best of our knowledge, our study is the first providing a thorough understanding and comparisons of VOC profiles obtained following the heating of sow fat as well as tainted and untainted boar fat at two elevated temperatures (150 and 180 °C) and sampling and analysis by solid phase microextraction gas chromatography–mass spectrometry (SPME-GC-MS).

## 2. Materials and Methods

### 2.1. Samples

Sow back fat (*n* = 6), tainted (*n* = 7) and untainted boar fat (*n* = 7) were collected from a local slaughterhouse. Sow fat was randomly selected. Tainted and untainted boar fat, on the other hand, were selected after these had been checked for boar taint by a trained assessor through an online human nose detection method (soldering iron). The collected samples were frozen at −20 °C at the slaughterhouse, transported in a cooler, and stored again at −20 °C. The presence or absence of boar taint was confirmed through the quantification of skatole and androstenone in fat by high-performance liquid chromatography fluorescence detection (HPLC-FD), which is described later in this section.

### 2.2. Chemicals

Methanol (CAS n° 67-56-1, HPLC grade, Sigma-Aldrich, Darmstadt, Germany), dansylhydrazine (CAS n° 33008-06-9, Sigma-Aldrich, Darmstadt, Germany ), boron trifluoride (BF_3_) at 20% in methanol *v/v* (CAS n° 373-57-9, VWR, Darmstadt, Germany), phosphoric acid (H_3_PO_4_) (CAS n° 7664-38-2, Sigma-Aldrich, Darmstadt, Germany), acetonitrile (CAS n° 75-05-8, HPLC grade, Supelco, Darmstadt, Germany), tetrahydrofuran (CAS n° 109-99-9, HPLC grade, Supelco, Darmstadt, Germany), liquid nitrogen (CAS n° 7727-37-9, Nippon Gases, Schoten, Belgium), 2,3-dimethylindole (CAS n°91-55-4, Sigma Aldrich, Darmstadt, Germany), skatole (CAS n° 83-34-1, Sigma Aldrich, Darmstadt, Germany), and androstenone (CAS n° 18339-16-7, Sigma Aldrich, Darmstadt, Germany ) were used in this experiment.

### 2.3. Skatole and Androstenone Quantification in Back Fat

This analysis allowed quantifying the skatole and androstenone content in both tainted and untainted boar fat samples. Boar fat is considered tainted if skatole concentrations are above the thresholds of 200 ng g^−1^ of fat and/or above 1000 ng g^−1^ for androstenone. These thresholds were selected given that the commonly accepted threshold generally range from 200 to 250 ng g^−1^ of fat for skatole and 500 to 1000 ng g^−1^ for androstenone [35]. Quantification of these molecules in back fat was performed on the basis of a method by Hansen-Moller (1994) [36], which consists of a methanolic extraction of the molecules, derivatization of androstenone, and analysis by high-performance liquid chromatography fluorescence detection (HPLC-FD). This protocol was slightly adapted as described in this section.

#### 2.3.1. Extraction of Androstenone and Skatole

Two mL of methanol was added to 0.50 g of back fat cut into pieces (0.5 cm square). The sample was homogenized by an Ultra-Turrax T25 (Janke & Kunkel, Straufen, Germany) for 30 s at 13,500 rpm. Then, 500 µL of methanol was added, and the sample was homogenized again for 30 s with the Ultra-Turrax; finally, 500 µL of methanol was added and homogenized for 1 min with the Ultra-Turrax. The sample was ultrasonicated for 5 min and placed in an ice bath for 15 min before centrifugation at 7700 rpm at 4 °C. Then, the supernatant was passed through a 0.45 µm filter paper (Whatmann, Darmstadt, Germany), and 140 µL was put in vial for analysis.

#### 2.3.2. Derivatization

The autosampler was programmed to mix 30 µL of 2% dansylhydrazine in methanol, 4.4 µL of water, and 10 µL of 20% *v/v* BF_3_ with 140 µL of methanolic extract. A reaction time of 5 min was observed; then, 20 µL of the incubated sample was injected into HPLC.

#### 2.3.3. High-Performance Liquid Chromatography Fluorescence Detection (HPLC-FD)

The analysis was performed by HPLC (1260 Infinity, Agilent Technologies, Santa Clara, CA, USA) with a kinetex column EVO C18 100 A (150 × 3.0 mm × 5 µm, Phenomenex, Utrecht, Belgium) and a precolumn AJO-9297, EVO C18 (Phenomenex, Utrecht, Belgium). The solutions for the mobile phase are prepared as follows: (A) H_3_PO_4_/deionized water (1:1000 *v/v*); (B) acetonitrile; (C) THF/deionized water (99:1 *v/v*). The elution gradient profile runs as presented in Table 1. The mobile phase was pumped at a flow rate of 0.5 mL min^−1^ throughout the process.

The detection with a fluorescence detector (FD) (Agilent Infinity 1260) was performed with an excitation wavelength of 285 nm and emission wavelength of 340 nm for skatole and 346 nm for excitation and 521 nm for emission of androstenone. The wavelength change takes place after 12 min of elution.

#### 2.3.4. Quantification of Skatole and Androstenone

Quantification of skatole and androstenone was made possible with matrix-matched calibration curves. These were prepared with sow fat (very low concentrations in skatole and absence of androstenone) that had been previously spiked with standards solutions. Calibrations curves were prepared for concentrations ranging from 45 to 500 ng/g for skatole and from 240 to 5000 ng/g for androstenone.

### 2.4. Analysis of VOCs Found in the Headspace of Heated Back Fat Samples

VOC profiles were established following 6 different analyses (i.e., 6 modalities): heating of sow fat at 150 °C, untainted boar fat at 150 °C, tainted boar fat at 150 °C, sow fat at 180 °C, untainted boar fat at 180 °C, and tainted boar fat at 180 °C. The analyses were performed as described in this section.

#### 2.4.1. Sample Preparation

First, 2.5 g of back fat was cut and then cooled by adding liquid nitrogen (−196 °C). The sample is ground for 5 s with an A11 basic IKA analytical grinder. Before recovering 1.0 g of sample in a vial, liquid nitrogen is added to the sample to freeze it. The sample is stored at −20 °C until analysis.

#### 2.4.2. SPME-GC-MS VOCs Analysis

Before proceeding to the headspace solid phase microextraction GC-MS analysis (HS-SPME-GC-MS), 1 µL of 2,3-dimethylindole at 125 µg mL^−1^ in methanol is added on the inside of the 20 mL vial, which is immediately sealed with a magnetic screw cap with a PTFE septum (Sigma-Aldrich, Darmstadt, Germany).

Incubation of the sample takes place at 150 °C (for the first analysis) or 180 °C (for the second analysis) for 20 min in a heated agitator (Gerstel, Mülheim an der Ruhr, Germany). Then, sampling of VOCs was achieved with a divinylbenzene/carboxen/polydimethylsiloxane (DVB/CAR/PDMS, 50/30 µm) SPME fiber (Supelco, Darmstadt, Germany) through a 5 min exposition in the headspace. The vials were shaken at 250 rpm (agitator on/off time: 10 s/1 s) during incubation and extraction. Desorption of the extracted and captured VOCs takes place for 2 min. Injection was performed in splitless mode at 270 °C. The fiber was conditioned for 20 min at injection temperature. Analyses were performed by GC-MS (7890A-5975C, Agilent Technologies, Santa Clara, CA, USA ) equipped with an HP-5 MS capillary column (30 m × 250 µm × 0.25 µm, Agilent Technologies, Santa Clara, CA, USA). Helium was used as a carrier gas at a flow rate of 1.2 mL/min. The oven temperature program was as follows: starting at 40 °C with a hold for 3 min; then, there is an increase of 5 °C/min up to 300 °C with a hold for 5 min. The mass spectrometer was set to have a temperature of 230 °C at the ion source and 150 °C at the quadrupole. The mass spectrometer was programmed with a SCAN/SIM acquisition mode. In SIM mode, the targeted ions were (quantitative ions in bold): 77, 103, and **130** for skatole; 130 and **144** for 2,3-dimethyl-indole; and lastly, 239, 257, and **272** for androstenone. The SIM mode allowed for semi-quantification of skatole and androstenone in the headspace using the following formula:

Target (ppb) = (area _target quant. ion/_area _I.S. quant. ion_) × mass of I.S. × (1/vial volume) × correction factor. (1)

The correction factor corresponds to 2.5 for skatole and 1/34 for androstenone; IS corresponds to the internal standard, i.e., 2,3-dimethylindole in this case.

In SCAN mode, mass spectra were scanned from 35 to 500 amu. Then, component identification was performed by comparison of the obtained spectra with mass spectra in a reference database (NIST17). Additionally, experimental retention indices (RI) were calculated following the injection of a mixture of n-alkanes C_8_-C_30_ (Sigma Aldrich, Darmstadt, Germany) under the same chromatographic conditions as those previously mentioned. This allowed the comparison of these RI to literature RI. Lastly, pure standards were injected for skatole (CAS n° 83-34-1, Sigma Aldrich) and androstenone (CAS n° 18339-16-7, Sigma Aldrich, Darmstadt, Germany) to ensure identification [37,38,39].

### 2.5. Data Analysis

General VOC profiles were established through a chromatographic deconvolution process (Agilent MassHunter Unknowns Analysis), and chromatographic areas were obtained for each VOC. Then, these results were used in two different ways. In the first case, they were first reported as a percentage of the total chromatographic area to allow a general analysis. General linear models (GLMs) were performed on these data to validate some observations. Fat type was used as a fixed factor and incubation temperature was used as a covariate for GLM. In the second case, the chromatographic area data were mass-normalized, auto-scaled, and log-transformed (generalized logarithm transformation) to obtain a distribution of the variables closer to normal and make results more comparable. Then, a principal component analysis (PCA) as well as a heatmap were generated with these normalized data. One-way analyses of variance (ANOVAs) were performed on the normalized data of the top 25 contributors to the differences observed. The PCA and heatmap were carried out on metaboanalyst [40]. Pearson correlation coefficients were determined for the skatole and androstenone headspace and content concentrations data. These coefficients as well as GLMs mentioned earlier were established with the Minitab 19 software (Minitab Inc., State College, PA, USA). Headspace/content correlation plots for skatole and androstenone were performed on Excel (Microsoft Office 2016).

## 3. Results and Discussion

In this section, results concerning the analysis of VOC profiles obtained with the high incubation temperatures used, i.e., 150 and 180 °C, will first be examined. Fat can be heated even more for boar taint detection; however, lipid oxidation occurs at a greater extent in this case. Therefore, in this research, 150 and 180 °C were studied, as it seemed to be a compromise between high temperature for the extraction of skatole and androstenone and minimization of lipid oxidation and the creation of degradation products, which could potentially interfere with the detection of boar taint compounds and saturate the sensors in the case of e-noses. Thus, the detection of skatole and androstenone in the headspace will be examined in the next section.

### 3.1. VOC Profiles Generated through High-Temperature Incubation of Fat

#### 3.1.1. General Understanding of the Generated VOC Profiles

A total of 48 compounds were correctly identified overall in fat samples regardless of their taint (Table 2). The profiles are composed of a large diversity of molecules including, amongst others, alcohols, aldehydes, furanes, and pyridine derivatives. Although some common characteristics are observed between the six different types of profiles obtained, some differences are also observed. These mainly exist between the heating temperatures rather than between the fat types.

In fact, it can be observed from Table 2 that the major group of compounds identified is not the same at 150 and 180 °C. Aldehydes are the most abundant at 150 °C ranging from 40.09% of the total profile for tainted fats to 55.00% for sow fats compared to much lower percentages of aldehydes at 180 °C, ranging from 19.09% for untainted fat to 26.81% for tainted fats (effect of temperature: *p*-value < 0.05). Amongst these aldehydes, some are present in much greater quantities compared to others. These include (*E*)-Dec-2-enal, (*E*)-Undec-2-enal, (*E*,*E*)-Hepta-2,4-dienal, and (*E*,*E*)-Deca-2,4-dienal, the latter accounting for up to 16.34% of the total profile in the case of sow fat.

On the other hand, the fatty acids group is the most present at 180 °C, making up 52.88% to 63.14% of the total profile at this temperature. Three fatty acids stand out: octadec-9-enoic acid (up to 31.93% of the total profile), hexadecanoic acid (up to 15.85%), and lastly, octadecanoic acid (up to 20.7%). Finding these three molecules as the most abundant fatty acids is in accordance with what has been found by Zhao et al. (2017) [41], who analyzed VOCs of stewed pork broth by solvent-assisted flavor evaporation (SAFE) combined with GC-MS.

Additionally, observing octadec-9-enoic acid and hexadecanoic acid as two of the three major fatty acids in the VOCs profile corresponds to the actual fatty acids content of back fat. In fact, several studies have analyzed the fatty acid composition of back fat and have found that the most abundant was octadec-9-enoic acid followed by hexadecanoic acid [42,43]. The hydrolysis of triglycerides into free fatty acids (FFAs) and glycerol is controlled by two main lipolytic enzymes: adipose triacylglycerol lipase (ATGL) regulating the hydrolysis of triacylglycerols into diacylglycerols and FFAs and hormone-sensitive lipase (HSL) regulating that of diacylglycerols into monoacylglycerols, FFAs, and glycerols [44]. Therefore, this explains the presence of FFAs in back fat.

Regarding their presence in the headspace, one must remember that such long-chain fatty acids possess low vapor pressures (e.g., octadec-9-enoic acid has a vapor pressure of 5.46 × 10^−7^ mm Hg at 25 °C [45]); therefore, greater incubation temperatures lead to greater headspace concentrations of these FFAs. With temperatures increasing from 150 to 180 °C, it can be seen from the data that the total acids found in the headspace increase for all three fat types (*p* < 0.05).

Serra et al. (2004) [42] and Rius et al. (2005) [34] who have also analyzed VOCs obtained following incubation of fat observed that aldehydes were the most abundant class of molecules, making up respectively 37.1% and 69.61% of the total VOC profiles. However, lower incubation temperatures (60 and 120 °C) were used in their study, which could explain the smaller volatilization of FFAs and hence the smaller relative abundance of these in their VOC profiles. Seeing that the total aldehydes percentage in the 180 °C profiles is lower is simply due to the fact that more volatiles are being released at 180 °C compared to 150 °C.

As observed in Table 2, the majority of aldehydes present are unsaturated, which is explained by the higher proportions of unsaturated fatty acids than saturated fatty acids in pork back fat [46]. The most abundant aldehydes are (*E*,*E*)-deca-2,4-dienal and (*E*,*E*)-hepta-2,4-dienal, which are VOCs produced following the oxidation of linoleic acid and linolenic acid, respectively, and which are known to have a fatty and fried smell [24,41,47,48]. Benzaldehyde has also been found to originate from linolenic acid degradation [49].

In smaller proportions are ketones and alcohol. The alcohols detected at these temperatures correspond to those that have been found by Rius et al. (2005) [34] at 120 °C. On the other hand, two of the three ketones (pentadecan-2-one and heptadecan-2-one) observed in our study have not been observed by the latter. However, Zhao et al. (2017) [41] have found pentadecan-2-one as part of the VOCs found in pork broth.

Furans have also been found in the profiles. Furans are well-known to be responsible for the characteristic odor of fried foodstuffs. These molecules are found in a multitude of food products, including meat products [50,51].

#### 3.1.2. Understanding the Differences between the VOC Profiles Generated

**Figure 1:**
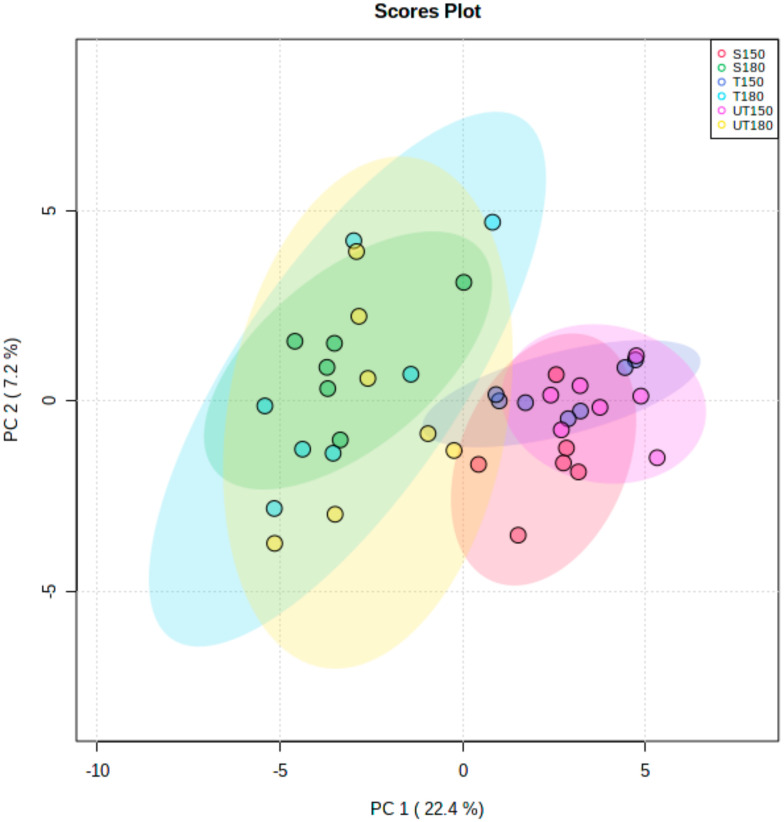
Principal component analysis (PCA) scores plot of component area normalized data of VOC profiles. Red and green dots indicate VOC profiles obtained for sow fat heated at 150 °C (*n* = 6) and 180 °C (*n* = 6), dark blue and light blue represent VOC profiles for tainted boar fat heated at 150 °C (*n* = 7) and 180 °C (*n* = 7); lastly, pink and yellow dots represent untainted fats at 150 °C (*n* = 7) and 180 °C (*n* = 7) respectively.

**Figure 2:**
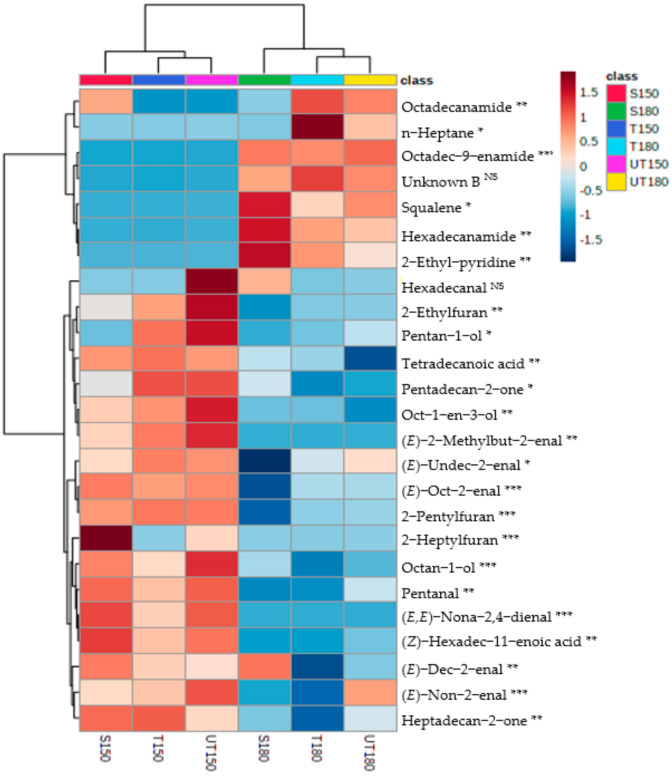
Heatmap generated with normalized data for the top 25 molecules responsible for the differences between the profiles. Each column corresponds to a studied modality. Red and green squares indicate VOC profiles obtained for sow fat heated at 150 and 180 °C, dark blue and light blue represent VOC profiles for tainted boar fat heated at 150 and 180 °C, and lastly, pink and yellow dots represent untainted fats at 150 and 180 °C, respectively. Results of ANOVAs are represented after the molecule name: ^NS^ indicates a *p*-value > 0.05 while *, **, *** indicate *p*-values < 0.05, <0.01, and <0.001 respectively.

Principal component analysis (PCA) was used to better visualize existing differences or groupings between the samples analyzed. Figure 1 represents a PCA scores plot of the first two principal components (PCs) of the VOC profiles dataset. Therefore, in this PCA scores plot, each sample analyzed is represented based on their respective VOC profiles. The first principal component (PC 1) explains 22.4% of the variation in the dataset, while the second principal component (PC 2) explains 7.2% of the variation. In this figure, the samples that are close to each other have similar VOC profiles. Therefore, the clear overlap of sow fat, tainted boar fat, and untainted boar fat respectively at 150 and 180 °C suggests that no net distinction is perceived between the VOC profiles of these three fat types. However, although a slight overlap is observed between the samples at 150 °C and those at 180 °C, a separation exists between the VOC profiles obtained following fat incubation at 150 and 180 °C. This suggests, as expected, that temperature has an impact on the generated VOCs. The molecules majorly responsible for the differences observed between the temperatures are described later in this section (Figure 2).

As a reminder, the general VOC profiles have been established based on an untargeted approach following the detection of molecules in SCAN mode. Hence, skatole and androstenone semi-quantified following SIM mode detection (addressed in the next section) have not been included in the PCA data. Several other molecules have been suggested in the literature as responsible for boar taint. These include indole, 4-phenyl but-3-en-2-one, styrene, 1,4-dichlorobenzene, 2-aminoacetophenone, 5-α-androst-16-en-3-α-ol, and 5-α-androst-16-en-3-β-ol [2,34,52,53]. However, these molecules were not observed in the SCAN data, and no targeted approach (such as the use of the SIM mode) was used to attempt to detect them. Hence, this partially explains the overlapping of tainted and untainted fats. Additionally, although these molecules are not detected here in SCAN mode due to very low analytical concentrations, these still impact sensory evaluation as they may be detected by the human. The concept of odor activity values (OAVs) is very important in such analysis. This one considers the concentration of a compound in the food matrix and its odor threshold. OAV values greater than 1 are considered to be main contributors to the total flavor [54,55]. The OAV in fat of several molecules introduced above have been studied by Gerlach et al. (2018) [56]. For example, they have found that androstenone has an OAV of 25 and skatole has an OAV of 40 in boar fat. On the other hand, (*E*,*E*)-deca-2,4-dienal, the most present aldehyde in our study, only had an OAV of 1. This suggests that although this molecule is present in high concentrations in our study (Table 2), it only minorly impacts sensory evaluation compared to boar taint compounds.

The interpretation of the molecules responsible for the difference between the two temperatures is eased through the elaboration of a heatmap (Figure 2). The level of significance of the differences observed can be observed after the molecule name. From the latter, it appears that significant differences exist for 23 of the 25 molecules most responsible for the differences perceived.

As mentioned earlier and as confirmed by this figure, it can be noticed that overall, the differences mainly reside between profiles at the different temperatures. The VOCs’ intensities are very different from one temperature to another and imply that assessors performing sensory evaluation at different temperature are not confined to the same working environment. This could lead to different results for the same sample. This stresses the importance of standardizing sensory evaluation protocols, from the training of the assessors to the evaluation per se performed in the slaughterhouse [27,57,58].

Some molecules are present in significantly higher concentrations in the headspace of fat heated at 150 °C compared to 180 °C. For example, this is the case for the aldehydes such as (*E*)-non-2-enal, (*E*)-undec-2-enal, and (*E*,*E*)-nona-2,4-dienal. As mentioned earlier, these molecules are secondary oxidation products of fatty acids. In meat, these molecules can further react. For example, the molecule (*E*,*E*)-deca-2,4-dienal can react with ammonia to produce 2-pentyl-pyridine [46]. Ammonia usually originates from the Strecker degradation of cysteine, which is an amino acid frequently found in the meat [59]. Careful attention was paid when sampling the fat before homogenization; however, the potential traces of muscle (Longissimus dorsi) in the sample cannot be excluded. The acceleration of such a reaction at high temperatures could explain the smaller headspace concentrations of (*E*,*E*)-deca-2,4-dienal in samples at 180 °C (Figure 2).

To the exception of octadec-9-enamide in the specific case of sow fat, the fatty amides, hexadecanamide, octadecanamide, and octadec-9-enamide, which are present in the 180 °C profiles, are simply absent from the profiles at 150 °C. Such amides have been obtained in several studies on the pyrolysis of meat products for waste management [60,61], hence demonstrating the implication of high temperatures in their production. These molecules are not simple degradation products of fatty acids and suggest once again the presence of small concentrations of proteins in the fat sample [60].

Another molecule present only in profiles at 180 °C is squalene. This one also has a low vapor pressure (6.3 × 10^−6^ mmHg at 25 °C), which explains its presence only at the higher temperature. Finding squalene in the three types of fats at this temperature is explained by the fact that squalene affects cholesterol production, which in turn affects the production of the steroid pregnenolone. All steroids and hence both androgens and estrogens in male and female pigs are produced starting from pregnenolone [53].

### 3.2. Detection of Skatole and Androstenone in the Headspace of Tainted and Untainted Boar Fat

**Figure 3:**
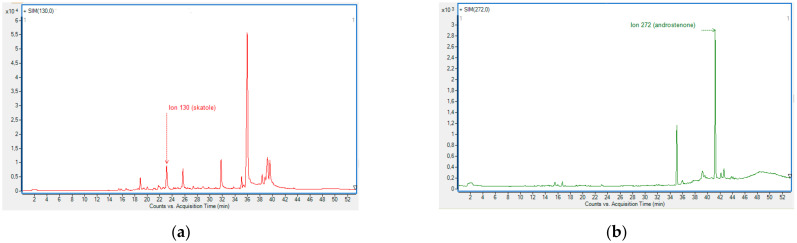
Detection of ions in selection ion monitoring mode (SIM mode) for samples incubated at 150 °C. Quantitative ions for semi-quantification of (**a**) skatole (*m/z* 130, peak at Rt = 23.096 min) and (**b**) androstenone (*m/z* 272, peak at Rt = 41.233 min).

Detection of the ions used for both qualification and semi-quantification of skatole and androstenone in boar fat was possible at both temperatures (Figure 3).

**Figure 4:**
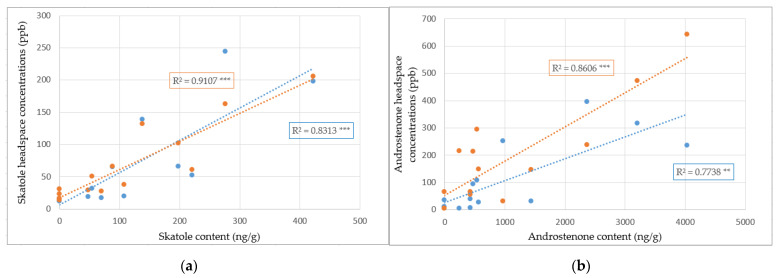
Correlation plots between headspace concentrations (ppb) and content concentrations (ng/g) of skatole (**a**) and androstenone (**b**) respectively at 150 °C (in blue, *n* = 7) and 180 °C (in orange, *n* = 7). For the content, values below the linearity range were set at 0 arbitrarily. Pearson correlation coefficients as well as their significance levels are represented on the graph. **, and *** indicate *p*-values < 0.01, and <0.001, respectively.

Additionally, positive correlation coefficients higher than 0.77 are observed between the content (Table S1 for skatole and androstenone back fat content) and headspace concentrations of skatole and androstenone at both 150 and 180 °C (Figure 4).

It can be observed from the skatole correlation plots that trends between content and emissions are similar at 150 and 180 °C, which therefore suggests that the skatole extraction yield and subsequently the concentrations perceived in the headspace are the same at these temperatures. To increase the headspace concentrations in skatole, several solutions exist. Amongst these solutions, a large increase in temperature, a reduction of the headspace volume, or simply the heating of bigger samples (that hence have a greater absolute quantity in skatole) could be considered. However, as mentioned earlier, in both cases, the VOCs profile will be rich in many other molecules, which might affect the response of the boar taint detection method used (saturation of the assessor’s nose in the case of sensory evaluation and sensor drift for sensor-based methods).

Different results appear for androstenone correlation plots. In fact, it can be seen from Figure 4b that more androstenone is emitted with increasing temperature. This can in part be explained by the fact that androstenone has a lower vapor pressure and hence a smaller tendency to volatilize compared to skatole, thus leading to better androstenone extractions at higher temperatures.

Low headspace concentrations (maximum below 250 ppb for skatole and 700 ppb for androstenone) could in part be explained by the strong matrix effects observed with boar fat. Given the lipophilic character of skatole and androstenone, an efficient extraction process is often used prior to analytical determinations of skatole and androstenone. Sample preparation usually begins with a heating or homogenization step followed by an extraction and purification step prior to analysis. Additionally, measurements are often performed based on liquefied fat from which connective tissues have been removed (only 60% of the adipose tissue is constituted of fat per se) [62]. Various methods have been developed in the last decade to quantify skatole and androstenone content based on headspace analysis. As it is the case in our study, these researchers have only incubated fat at high temperatures prior to quantification of the boar taint compound. However, to compensate for matrix effects and subsequent low analyte extraction, internal standards were spiked directly in the liquefied fat [63,64]. This procedure was not performed in our study, as we wanted to determine real headspace concentrations of skatole and androstenone, justifying the injection of the internal standard 2,3-dimethylindole directly in the headspace of the vial. It is important to note that what is perceived by the sensory assessor in the slaughterhouse or by the consumer when eating pork are compounds that are present in gaseous form in the headspace. In fact, VOCs can reach the nasal cavity either through orthonasal olfaction (direct inhalation in front of the nose) or through the throat while chewing (retronasal olfaction) [65].

The positive and significant Pearson correlation coefficients between the headspace concentration and the content for skatole and androstenone indicate that what is found in the headspace at both temperatures is a good representation of boar taint in the fat. Although extraction is similar for skatole at 150 and 180 °C (typical cooking temperatures of pork), the greater headspace concentrations for androstenone at 180 °C imply that higher temperatures allow a better representation of boar taint. One must remember that boar taint is a complex smell composed of a large variety of molecules. Only 33% of boar taint is explained by skatole alone, while 50% of the taint is explained by the combination of skatole and androstenone [66]. Whether it is for sensory evaluation or for sensor-based methods focusing on the detection of skatole and androstenone, using higher temperatures to detect greater amounts of androstenone should allow a better visualization of boar taint as a whole. Lastly, the headspace concentrations are low for sensor-based methods (which often operate in up to the ppm [67]) and hence emphasize the importance of testing even higher temperatures.

## 4. Conclusions

To the best of our knowledge, this study is the first analyzing VOCs emitted by back fat samples when heated at elevated temperatures. The aim of this study was to perform a general analysis of VOC profiles obtained with fat presenting different boar taint intensities, but being an exploratory study, it did not intend to rigorously compare the impact of different taint combinations on the emitted VOCs. As a reminder, the comprehension of the VOC profiles at these typical cooking temperatures was primordial to understand what composes the exact smell perceived during the sensory evaluation of boar taint at these temperatures and secondly understand whether VOCs sensor-based methods for boar taint detection at these temperatures can be developed.

Great differences were observed between the VOC profiles depending on the incubation temperature. Different VOC profiles might result in differences in classification of the same tainted and untainted fats when heated at different temperatures. Therefore, this stresses the need to develop and use a standardized method for the sensory evaluation of boar taint.

VOCs sensors for skatole and androstenone detection could be developed for incubation temperatures of 150 and 180 °C given that both molecules are found in the headspace. However, the low headspace concentration observed for both these molecules should encourage further research into higher incubation temperatures. Analyses of the general VOCs headspace should always complement research into skatole and androstenone detection as the complexity of the VOCs profile might increase with temperature. The impact of fatty acids and aldehydes (as these are the most abundant in the VOC profiles at both temperatures) should be tested on sensor material to determine the rate at which sensor drift occurs to elaborate more robust drift correction algorithms and finally determine after how many analyses the sensors should be disposed of. Solutions to reduce the development of products from lipid oxidation, such as working in a closed and controlled environment, should be further looked into for sensor development.

## Figures and Tables

**Table 1:** Elution gradient for the separation of skatole and androstenone on an High-Performance Liquid Chromatography Fluorescence Detection (HPLC) system.

Time (min)	H3PO4/Deionized Water (1:1000 *v*/*v*)	Acetonitrile	THF/Deionized Water (99:1 *v*/*v*)
0	73	0	27
5.3	73	0	27
7.3	42	24	34
13	42	24	34
13.3	10	0	90
18	10	0	90
24	73	0	27
28	73	0	27

**Table 2:** GC-MS results of VOCs found in the headspace of heated fat. Results are expressed in relative abundance (%, mean ± s.d.). For each molecule, general information such as the match factor of the molecule when compared to the database, its CAS number, as well as the calculated RI and literature RI (NIST17) are given. Finally, relative abundance (%) is given for the six modalities tested: sow back fat heated at 150 °C (S 150 °C) and 180 °C (S 180 °C), untainted back fat heated at 150 °C (UT 150 °C) and 180 °C (UT 180 °C), as well as tainted back fat heated at 150 °C (T 150 °C) and at 180 °C (T 180 °C).

	Match Factor	CAS	Calculated RI	Litt. RI	S 150 °C (*n* = 6)	S 180 °C (*n* = 6)	UT 150 °C (*n* = 7)	UT 180 °C (*n* = 7)	T 150 °C (*n* = 7)	T 180 °C (*n* = 7)
Alcohol										
Pent-1-en-3-ol	87	616-25-1	712	671	0.00 ± 0.00	0.31 ± 0.38	0.2 ± 0.22	0.07 ± 0.18	0.32 ± 0.3	0.08 ± 0.14
Pentan-1-ol	91	71-41-0	769	761	0.14 ± 0.23	0.24 ± 0.46	0.69 ± 0.25	0.35 ± 0.43	0.93 ± 0.5	0.45 ± 0.63
Heptan-1-ol	86	111-70-6	968	960	0.06 ± 0.09	0.37 ± 0.57	0.07 ± 0.08	0.00 ± 0.00	0.04 ± 0.12	0.06 ± 0.15
Oct-1-en-3-ol	93	3391-86-4	978	969	0.25 ± 0.29	0.05 ± 0.11	0.6 ± 0.23	0.00 ± 0.00	0.54 ± 0.48	0.29 ± 0.75
Octan-1-ol	90	111-87-5	1070	1059	0.47 ± 0.29	0.31 ± 0.62	0.43 ± 0.21	0.11 ± 0.3	0.34 ± 0.27	0.00 ± 0.00
*Total alcohol*					0.91	1.28	1.99	0.53	2.17	0.87
Aldehydes										
3-Methylbutanal	92	590-86-3	701	650	0.00 ± 0.00	0.00 ± 0.00	0.00 ± 0.00	0.09 ± 0.17	0.05 ± 0.09	0.16 ± 0.27
Pentanal	94	110-62-3	719	707	0.71 ± 0.34	0.26 ± 0.64	0.88 ± 0.34	0.57 ± 0.73	0.67 ± 0.57	0.31 ± 0.82
(*E*)-2-Methylbut-2-enal	86	1115-11-3	759	692	0.11 ± 0.17	0.00 ± 0.00	0.2 ± 0.17	0.00 ± 0.00	0.23 ± 0.21	0.00 ± 0.00
Hexanal	98	66-25-1	795	806	2.22 ± 1.32	1.26 ± 1.89	2.47 ± 1.1	0.49 ± 0.67	3.28 ± 1.37	2.32 ± 1.86
(*E*)-Hex-2-enal	95	6728-26-3	848	814	0.14 ± 0.23	0.07 ± 0.18	0.26 ± 0.43	0.00 ± 0.00	0.12 ± 0.22	0.13 ± 0.25
Heptanal	96	111-71-7	898	905	0.25 ± 0.29	0.92 ± 2.03	0.4 ± 0.29	0.17 ± 0.22	0.45 ± 0.31	0.22 ± 0.28
(*E*)-Hept-2-enal	95	18829-55-5	954	913	4.45 ± 1.53	2.2 ± 1.62	3.59 ± 1.06	0.99 ± 1.27	3.76 ± 1.41	0.68 ± 0.89
Benzaldehyde	91	100-52-7	957	982	1.95 ± 1.19	0.45 ± 0.62	0.6 ± 0.84	0.31 ± 0.38	0.81 ± 0.51	0.58 ± 0.76
(*E*,*E*)-Hepta-2,4-dienal	93	4313-03-5	995	921	12.14 ± 4.58	3.13 ± 3.98	8.06 ± 2.32	6.48 ± 5.5	7.78 ± 1.6	6.21 ± 4.47
Octanal	91	124-13-0	1001	1005	0.15 ± 0.37	1.07 ± 2.62	0.35 ± 0.4	0.14 ± 0.24	0.41 ± 0.39	0.38 ± 0.53
5-Ethylcyclopent-1-enecarboxaldehyde	83	36431-51-3	1027	1020	0.07 ± 0.16	0.00 ± 0.00	0.12 ± 0.21	0.00 ± 0.00	0.05 ± 0.12	0.12 ± 0.31
Benzeneacetaldehyde	90	122-78-1	1042	1081	0.16 ± 0.1	0.01 ± 0.03	0.13 ± 0.07	0.08 ± 0.11	0.09 ± 0.09	0.07 ± 0.1
(*E*)-Oct-2-enal	81	2548-87-0	1057	1013	1.52 ± 0.7	0.00 ± 0.00	1.51 ± 0.6	0.37 ± 0.45	1.43 ± 0.36	1.71 ± 3.53
Nonanal	97	124-19-6	1102	1104	2.16 ± 0.86	1.71 ± 1.87	2.16 ± 0.71	1.96 ± 1.51	2.48 ± 0.69	1.85 ± 1.34
(*E*)-Non-2-enal	96	18829-56-6	1159	1112	0.52 ± 0.47	0.26 ± 0.4	0.56 ± 0.25	0.83 ± 0.79	0.4 ± 0.29	0.00 ± 0.00
(*E*,*E*)-Nona-2,4-dienal	93	5910-87-2	1213	1120	0.73 ± 0.31	0.00 ± 0.00	0.64 ± 0.35	0.00 ± 0.00	0.36 ± 0.27	0.00 ± 0.00
(*E*)-Dec-2-enal	97	3913-81-3	1261	1212	4.26 ± 1.77	3.47 ± 2.87	3.56 ± 1.28	1.9 ± 3.33	2.88 ± 0.96	2.16 ± 4.7
(*E*,*E*)-Deca-2,4-dienal	97	25152-84-5	1316	1220	16.34 ± 3.29	4.38 ± 4.27	15.59 ± 4.76	3.14 ± 2.44	10.47 ± 3.51	6.09 ± 4.18
(*E*)-Undec-2-enal	97	2463-77-6	1363	1311	7.12 ± 2.74	0.17 ± 0.41	6.03 ± 2.58	1.58 ± 1.34	4.4 ± 1.71	3.83 ± 5.07
Hexadecanal	96	629-80-1	1814	1800	0.00 ± 0.00	0.03 ± 0.06	0.26 ± 0.41	0.00 ± 0.00	0.00 ± 0.00	0.00 ± 0.00
*Total aldehydes*					55.00	19.37	47.37	19.09	40.09	26.81
Alkanes										
n-Heptane	88	142-82-5	719	717	0.00 ± 0.00	0.00 ± 0.00	0.00 ± 0.00	0.14 ± 0.24	0.00 ± 0.00	0.36 ± 0.62
*Total alkanes*					0.00	0.00	0.00	0.14	0.00	0.36
Furans										
2-Ethylfuran	87	3208-16-0	722	742	0.12 ± 0.2	0.00 ± 0.00	0.27 ± 0.15	0.03 ± 0.07	0.34 ± 0.35	0.02 ± 0.05
2-Pentylfuran	91	3777-69-3	990	1040	1.69 ± 0.54	0.2 ± 0.36	1.52 ± 0.63	3.79 ± 9.28	1.83 ± 0.63	1.1 ± 1.95
2-[(*E*)-pent-1-enyl]furan	85	81677-78-3	997	1048	0.00 ± 0.00	0.00 ± 0.00	0.04 ± 0.1	0.00 ± 0.00	0.09 ± 0.2	0.00 ± 0.00
2-Heptylfuran	88	3777-71-7	1190	1239	0.32 ± 0.32	0.00 ± 0.00	0.12 ± 0.23	0.00 ± 0.00	0.00 ± 0.00	0.00 ± 0.00
*Total furans*					2.13	0.2	1.95	3.82	2.26	1.12
Ketones										
Pentan-3-one	84	96-22-0	681	654	0.00 ± 0.00	0.05 ± 0.08	0.00 ± 0.00	0.00 ± 0.00	0.00 ± 0.00	0.01 ± 0.02
Pentadecan-2-one	95	2345-28-0	1697	1648	4.03 ± 1.12	1.46 ± 1.18	2.65 ± 1.57	1.92 ± 3.24	2.15 ± 1.05	0.7 ± 0.78
Heptadecan-2-one	93	2922-51-2	1900	1847	1.81 ± 0.58	0.45 ± 0.54	0.97 ± 0.71	1.24 ± 1.54	1.67 ± 1.17	0.26 ± 0.7
*Total ketones*					5.84	1.96	3.62	3.15	3.82	0.97
Acids										
Nonanoic acid	91	112-05-0	1276	1272	0.15 ± 0.37	0.00 ± 0.00	0.27 ± 0.48	0.00 ± 0.00	0.11 ± 0.28	0.16 ± 0.43
Tetradecanoic acid	98	544-63-8	1764	1769	1.44 ± 0.35	0.95 ± 0.91	2.25 ± 0.62	1.23 ± 2.19	2.53 ± 0.56	1.21 ± 1.36
(*Z*)-Hexadec-11-enoic acid	80	2271-34-3	1903	1886	1.44 ± 1.15	0.00 ± 0.00	2.03 ± 1.75	0.38 ± 1	2.11 ± 2.09	0.00 ± 0.00
Hexadecanoic acid	96	57-10-3	1976	1968	13.65 ± 6.53	15.23 ± 20.87	12.74 ± 4.68	9.82 ± 9.7	11.27 ± 3.41	15.85 ± 19.38
Octadec-9-enoic acid	83	112-79-8	2143	2133	14.46 ± 7.93	31.93 ± 20.34	21.08 ± 8.79	29.51 ± 27.02	29.24 ± 12.83	11.94 ± 13.1
Octadecanoic acid	88	57-11-4	2178	2167	2.59 ± 1.51	11.83 ± 9.51	3.26 ± 1.72	15.54 ± 22.13	3.15 ± 1.77	20.7 ± 30.62
Octadeca-9,12-dienoic acid	84	544-71-8	2283	2183	1.68 ± 0.92	3.2 ± 1.76	2.31 ± 2.34	3.56 ± 5.41	2.56 ± 2.63	3.03 ± 3.47
*Total acids*					35.41	63.14	43.94	60.03	50.97	52.88
Amides										
Hexadecanamide	92	629-54-9	2192	2021	0.00 ± 0.00	3.38 ± 2.99	0.00 ± 0.00	1.27 ± 1.71	0.00 ± 0.00	3.43 ± 4.05
Octadec-9-enamide	88	301-02-0	2361	2228	0.00 ± 0.00	3.46 ± 1.84	0.00 ± 0.00	3.92 ± 4.68	0.00 ± 0.00	3.05 ± 3.69
Octadecanamide	89	124-26-5	2387	2220	0.09 ± 0.1	0.14 ± 0.34	0.00 ± 0.00	2.05 ± 3.42	0.00 ± 0.00	1.4 ± 1.64
*Total amides*					0.09	6.97	0.00	7.24	0.00	7.88
Pyridine derivatives										
2-Ethyl-pyridine	89	100-71-0	902	887	0.00 ± 0.00	0.47 ± 0.42	0.00 ± 0.00	0.12 ± 0.21	0.00 ± 0.00	0.17 ± 0.28
2-Pentyl-pyridine	90	2294-76-0	1196	1185	0.43 ± 0.49	0.86 ± 0.72	0.49 ± 0.18	0.29 ± 0.45	0.11 ± 0.19	0.89 ± 0.91
*Total pyridines derivates*					0.43	1.34	0.49	0.41	0.11	1.06
Others										
Unknown A			662		0.00 ± 0.00	0.00 ± 0.00.01	0.00 ± 0.00	0.00 ± 0.00	0.02 ± 0.05	0.01 ± 0.02
Unknown B			1714		0.00 ± 0.00	1 ± 1.56	0.00 ± 0.00	1.14 ± 1.65	0.00 ± 0.00	3.78 ± 2.6
Unknown C			1868		0.2 ± 0.28	0.31 ± 0.6	0.44 ± 0.25	0.06 ± 0.17	0.38 ± 0.27	0.2 ± 0.38
gamma-Palmitolactone	90	730-46-1	2104	1980	0.00 ± 0.00	0.00 ± 0.00	0.2 ± 0.54	3.47 ± 5.32	0.19 ± 0.5	2.85 ± 3.82
delta-hexadecalactone	85	7370-44-7	2133	2000	0.00 ± 0.00	4.16 ± 10.18	0.00 ± 0.00	0.76 ± 2.02	0.00 ± 0.00	1.04 ± 2.76
Squalene	89	111-02-4	2830	2914	0.00 ± 0.00	0.27 ± 0.3	0.00 ± 0.00	0.15 ± 0.19	0.00 ± 0.00	0.18 ± 0.24
*Total others*					0.2	5.74	0.64	5.59	0.58	8.06
Total					100.00	100.00	100.00	100.00	100.00	100.00

## Data Availability

Data will be available upon request from the corresponding author.

## Supplementary Materials

The following are available online at https://www.mdpi.com/article/10.3390/foods10061311/s1, Table S1: Quantification of skatole and androstenone in fat determined by HPLC-FD. The mention < LR indicates that the content is below the linearity range (45 to 500 ng/g for skatole and 240 to 5000 ng/g for androstenone).
