# Supplementary material for: Comprehensive SPME-GC-MS Analysis of VOC Profiles Obtained Following High-Temperature Heating of Pork Back Fat with Varying Boar Taint Intensities
Source: Foods. 2021 Jun 7;10(6):1311. doi: 10.3390/foods10061311 (PMC8227496; doi:10.3390/foods10061311)
Supplement: Supplementary file 1 [file foods-10-01311-s001.zip › foods-1250086-supplementary.pdf]

Supplementary Materials

Table S1. Quantification of skatole and androstenone in fat determined by HPLC-FD. The mention < LR indicates that the content is below the linearity range (45 to 500 ng/g for skatole and 240 to 5000 ng/g for androstenone).

Sample Number	Fat Content (ng/g of Fat)	
	**Skatole**	**Androstenone**
	**Untainted fat**	
**1**	54.8	537.8
**2**	47.6	569.8
**3**	107.5	423.7
**4**	<LR	<LR
**5**	<LR	<LR
**6**	69.7	245.9
**7**	88.9	429.7
	**Tainted fat**	
**1**	<LR	4036.8
**2**	<LR	1444.6
**3**	221.3	477.2
**4**	276.1	967.9
**5**	422.9	<LR
**6**	198.1	3208.3
**7**	138.2	2370.0
